# Autophagy in the Thymic Epithelium Is Dispensable for the Development of Self-Tolerance in a Novel Mouse Model

**DOI:** 10.1371/journal.pone.0038933

**Published:** 2012-06-18

**Authors:** Supawadee Sukseree, Michael Mildner, Heidemarie Rossiter, Johannes Pammer, Cheng-Feng Zhang, Ramida Watanapokasin, Erwin Tschachler, Leopold Eckhart

**Affiliations:** 1 Research Division of Biology and Pathobiology of the Skin, Department of Dermatology, Medical University of Vienna, Vienna, Austria; 2 Department of Biochemistry, Faculty of Medicine, Srinakharinwirot University, Bangkok, Thailand; 3 Institute of Clinical Pathology, Medical University of Vienna, Vienna, Austria; 4 Department of Dermatology, Huashan Hospital, Fudan University, Shanghai, People’s Republic of China; Juntendo University School of Medicine, Japan

## Abstract

The thymic epithelium plays critical roles in the positive and negative selection of T cells. Recently, it was proposed that autophagy in thymic epithelial cells is essential for the induction of T cell tolerance to self antigens and thus for the prevention of autoimmune diseases. Here we have tested this hypothesis using mouse models in which autophagy was blocked specifically in epithelial cells expressing keratin 14 (K14), including the precursor of thymic epithelial cells. While the thymic epithelial cells of mice carrying the floxed *Atg7* gene (ATG7 f/f) showed a high level of autophagy, as determined by LC3 Western blot analysis and fluorescence detection of the recombinant green fluorescent protein (GFP)-LC3 reporter protein on autophagosomes, autophagy in the thymic epithelium was efficiently suppressed by deletion of the *Atg7* gene using the Cre-loxP system (ATG7 f/f K14-Cre). Suppression of autophagy led to the massive accumulation of p62/sequestosome 1 (SQSTM1) in thymic epithelial cells. However, the structure of the thymic epithelium as well as the organization and the size of the thymus were not altered in mutant mice. The ratio of CD4 to CD8-positive T cells, as well as the frequency of activated (CD69+) CD4 T cells in lymphoid organs, did not differ between mice with autophagy-competent and autophagy-deficient thymic epithelium. Inflammatory infiltrating cells, potentially indicative of autoimmune reactions, were present in the liver, lung, and colon of a similar fraction of ATG7 f/f and ATG7 f/f K14-Cre mice. In contrast to previously reported mice, that had received an autophagy-deficient thymus transplant, ATG7 f/f K14-Cre mice did not suffer from autoimmunity-induced weight loss. In summary, the results of this study suggest that autophagy in the thymic epithelium is dispensable for negative selection of autoreactive T cells.

## Introduction

Autophagy is an evolutionarily conserved process in which the cell degrades its own components. It is critical for the intracellular quality control of proteins, the maintenance of metabolism during starvation, cellular renovation during development and differentiation as well as for anti-bacterial and anti-viral defense [1]–[3]. Macroautophagy is considered the predominant mode of autophagy in mammalian tissues [2] and will hereafter be referred to as “autophagy”. Chaperone-mediated autophagy and microautophagy are alternative mechanisms of autophagy that mediate the degradation of different subcellular substrates in a largely non-redundant manner [2],[4].

Autophagy is controlled by a defined set of evolutionarily conserved genes which have been reviewed extensively [2], [5], [6]. *Atg5* and *Atg7* are among the best characterized autophagy-related genes as their protein products play essential roles in a key step of autophagy, i.e. the conversion of the cytosolic form of microtubule-associated protein light chain 3 (LC3), LC3-I, into the lipidated form, LC3-II. The latter binds to the isolation membrane of the forming autophagosome and interacts with p62/SQSTM1, an adaptor protein that targets cytoplasmic proteins for selective degradation [2], [6]. Hence, inactivation of *Atg5* or *Atg7* inhibits autophagosome formation and leads to accumulation of LC3-I and p62 which can be monitored by immunolabeling [7]. A reporter system for the visualization of autophagy in tissues has been established by the recombinant fusion of LC3 to GFP so that autophagosomes are labeled by green fluorescence [8]. Targeted knockout of *Atg5* or *Atg7* in the mouse led to perinatal lethality [9], [10] whereas tissue-specific deletions of either one of the two genes resulted in viable mice with distinct defects, thus revealing roles of autophagy in development, cell differentiation and molecular processes [11]. The phenotypes of mice carrying deletions of *Atg5* and *Atg7* were essentially identical [11].

Recently, Nedjic et al. have suggested a novel function of autophagy in the thymus [12]. Thymic epithelial cells (TECs) show a high level of constitutive, starvation-independent autophagy [8]. To investigate the biological significance of this process, Nedjic et al. transplanted the thymus of ATG5-deficient embryos under the kidney capsule of autophagy-competent adult mice. In comparison to thymus grafts from normal embryos, thymi of autophagy-deficient embryos remained smaller but developed a normal structural organization into a cortex and a medulla. When thymi were grafted into athymic (nude) mice, the recipients of ATG5-negative thymi developed a higher frequency of activated CD4 T cells than recipients of ATG5-positive thymi. These mice had enlarged lymph nodes, flaky skin, atrophy of the uterus, absence of fat pads, and an enlarged colon [12]. Moreover, the colon, liver, lung, uterus and Harderian glands of mice receiving an autophagy-deficient thymus showed massive inflammatory infiltrates on thin sections stained by hematoxylin and eosin (H&E). Mice bearing an ATG5-negative thymus started to lose weight approximately 1 month after grafting and later had to be killed because of severe autoimmune disease. Together with another study demonstrating that autophagic compartments gain access to the MHC class II compartments in thymic epithelium [13], these data have established the concept of autophagy-dependent antigen processing in TECs [14], [15]. Nedjic et al. suggested that this process is essential for negative selection of T cells and for the development of self tolerance [12].

Here, we have tested the hypothesis put forward by Nedjic et al. in an alternative model [12]. Autophagy in thymic epithelium was suppressed by genetic deletion of ATG7 in epithelial cells. To this end, mice carrying a floxed *Atg7* gene [10] were crossed with mice expressing the Cre recombinase under the control of the keratin 14 (K14) promoter. This promoter is active in epithelial stem cells and is therefore routinely used to achieve the deletion of floxed genes in epidermal keratinocytes. However, the K14 promoter is also active in the progenitor cells of the thymic epithelium [16]. These progenitor cells give rise to the cortical and medullary epithelium both in embryonic [17] and postnatal [16] development of the thymus. Accordingly, ATG7 was deleted both in the epidermis and in the thymic epithelium of our mouse model.

We show that our experimental approach leads to the suppression of autophagy in the endogenous thymic epithelium. Mice deficient in autophagy within TECs developed normally without significant signs of autoimmunity. Thus, our study calls for a revision of the previously proposed role of autophagy in the establishment of self-tolerance.

## Results

### Deletion of ATG7 in K14-expressing Cells Suppresses Constitutive Autophagy in the Thymic Epithelium

**Figure 1:**
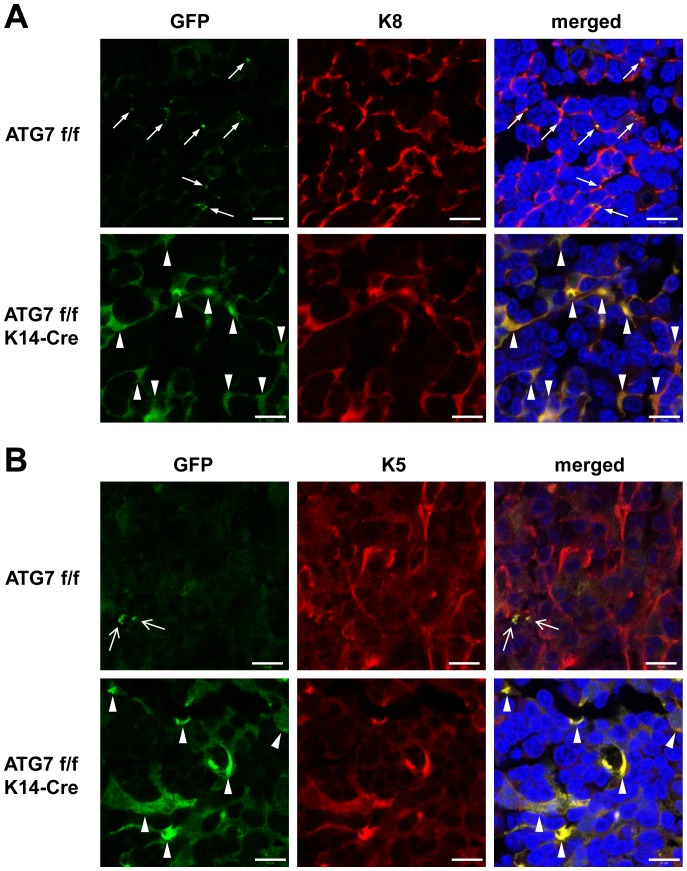
Deletion of ATG7 in K14-expressing cells suppresses autophagy in the thymic epithelium of GFP-LC3 transgenic mice. Thymi from GFP-LC3 ATG7 f/f and ATG7 f/f K14-Cre were cryo-sectioned and immunolabeled with anti-keratin 8 (K8) (**A**) and anti-K5 antibodies (red) (**B**). The immunolabeling and the green fluorescence of the GFP-LC3 fusion protein were viewed under a confocal laser scanning microscope. Regions of the thymus cortex (**A**) and medulla (**B**) are shown. Arrows in A point to GFP-LC3 puncta corresponding to labeled autophagosomes. Differently shaped arrows point to green fluorescent dots of uncertain identity that were detected at low frequency in the medulla (**B**). Arrowheads in **A** and **B** indicate diffuse cytosolic accumulation of GFP-LC3 in the epithelial cells of the cortex (**A**) and the medulla (**B**). Bar, 10 µm.

ATG7-floxed mice [10] and K14-Cre mice were crossed to abolish the expression of ATG7 in all cells in which the K14 promoter is active, including the precursor of TECs [16]. Mice homozygous for the floxed ATG7 allele are referred to as ATG7 f/f and mice also carrying K14-Cre are referred to as ATG7 f/f K14-Cre. To investigate the blockade of autophagy in thymic epithelium *in vivo*, we generated an ATG7-floxed mouse line carrying the GFP-LC3 transgene comprising GFP-LC3 ATG7 f/f and GFP-LC3 ATG7 f/f K14-Cre mice. The GFP-LC3 protein allows the detection of autophagosomes by fluorescence microscopy of thin sections. In agreement with previous reports [8], [12], we detected GFP-LC3 puncta in autophagy-competent thymic epithelium (GFP-LC3 ATG7 f/f), indicating constitutive, starvation-independent autophagy (Fig. 1). GFP-LC3 puncta were highly abundant in the thymic cortical epithelium (Fig. 1A) whereas green fluorescent puncta were rare in the medullary epithelium (Fig. 1B). As recommended by Mizushima et al. [8], the rare green fluorescent puncta in the medulla were considered ambiguous with regard to an association with autophagosomes. Cre-mediated deletion of ATG7 (GFP-LC3 ATG7 f/f K14-Cre) abolished the formation of GFP-LC3 puncta and led to a diffuse cytoplasmic accumulation of fluorescence in the epithelia of both thymic compartments (Fig. 1A, B). This suggested that GFP-LC3 is subjected to degradation by ATG7-dependent autophagy in the cortical and medullary epithelium of K14-Cre-negative mice and that K14-Cre-mediated deletion of ATG7 f/f efficiently abrogated this autophagic flux in all TECs.

**Figure 2:**
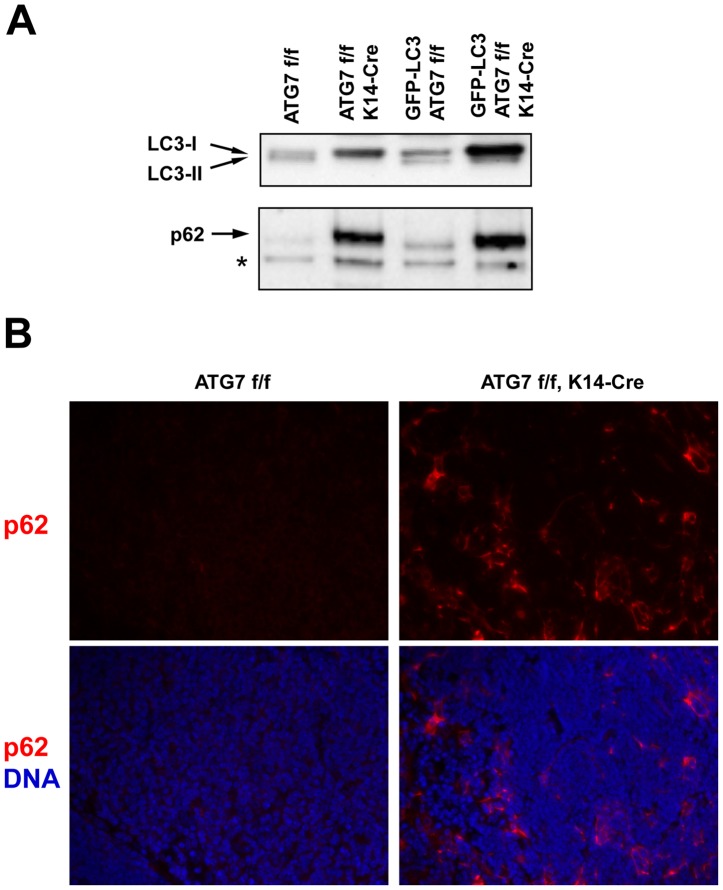
Deletion of ATG7 in K14-expressing cells suppresses autophagy and leads to accumulation of p62 in the thymus. (**A**) Thymus lysates of the ATG7 f/f and ATG7 f/f K14-Cre, either carrying the GFP-LC3 transgene or not, were subjected to Western blot analysis for LC3 (upper panel) and p62 (lower panel). Bands corresponding to LC3-I and LC3-II (indicative of active autophagy) are marked by arrows. Note the presence of LC3-II bands in ATG7 f/f thymi and the accumulation of LC3-I as well as p62 in ATG7 f/f K14-Cre mice. The position of an unspecific band, that shows equal loading of the lanes, is marked by an asterisk. (**B**) Immunofluorescence analysis of p62 expression (red) in the thymus. Note the accumulation of p62 in the characteristically shaped epithelial cells of ATG7 f/f K14-Cre mice. Nuclei are labeled with Hoechst 33258 (blue). The complete field of view under 200-fold magnification is shown in each panel.

Next, autophagy was monitored by Western blot analysis of LC3 and p62 in thymi of ATG7 f/f and ATG7 f/f K14-Cre mice. In agreement with the detection of constitutive autophagy in ATG7 f/f GFP-LC3 mice (Fig. 1), the LC3-II band was detected in protein lysates of these thymi. LC3-II was also present in the thymus of ATG7 f/f that were not crossed with GFP-LC3 transgenic mice (Fig. 2A). By contrast, thymus lysates of ATG7 f/f K14-Cre mice of both lines (GFP-LC3-negative or positive) did not contain LC3-II but showed massive accumulation of LC3-I, indicating the blockade of an otherwise constitutive conversion of LC3-I to LC3-II in the thymus. The suppression of autophagy was corroborated by a strong accumulation of p62 in the thymus of ATG7 f/f K14-Cre mice (Fig. 2A). Immunofluorescence analysis showed that p62 accumulated in the epithelial cells of the thymus (Fig. 2B).

### Genetic Suppression of Autophagy in Thymic Epithelium does not Compromise the Structure of the Thymus nor does it Lead to Severe Changes in Lymphocyte Differentiation

**Figure 3:**
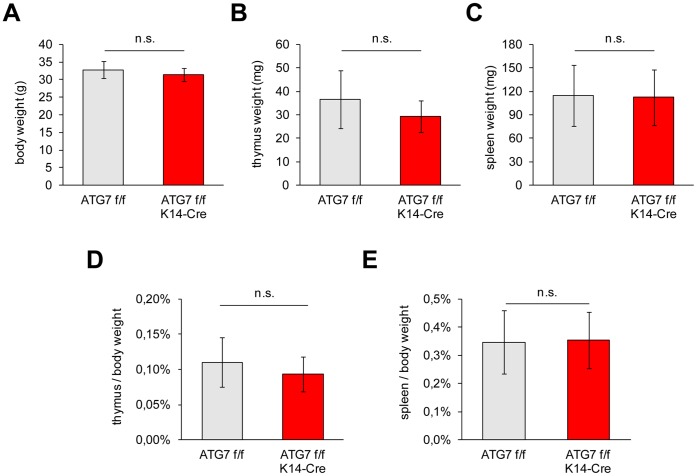
The weights of the body, thymus and spleen of ATG7 f/f K14-Cre mice are normal. The weights of the total body (**A**), the thymus (**B**) and the spleen (**C**) were determined for male mice in the age range from 6 to 12 months. Note that data for female mice are shown in figure S1. The weight data of mice expressing ATG7 (ATG7 f/f) (n = 14) or lacking ATG7 (ATG7 f/f K14-Cre) (n = 11) in the thymic epithelium were compared in absolute numbers or relative to the body weight (**D**, **E**). Statistical analysis using the t-test showed that there were only non-significant (n.s.) differences between the genotypes.

ATG7 f/f K14-Cre mice were viable and as fertile as their ATG7 f/f littermates. The body weights of the mice of both genotypes did not differ significantly (Fig. 3A), and individuals of ATG7 f/f and ATG7 f/f K14-Cre mice reached an age of more than 2 years. The weights of the thymus and of the spleen of aged mice did not differ between ATG7 f/f and ATG7 f/f K14-Cre mice, both in absolute terms and in relation to the body weight (Fig. 3B, C, D, E; Fig. S1 (female mice); and data not shown). Lymph nodes showed some variation in size among individuals of both genotypes, however there was no consistent difference between ATG7 f/f K14-Cre and control mice (not shown). H&E staining of thin sections of the thymus showed a normal structure in ATG7 f/f K14-Cre mice (Fig. S2A), and the expression pattern of K5 and K8 was also normal in thymus of ATG7 f/f K14-Cre mice as shown by immunofluorescence analysis (Fig. S2B).

**Figure 4:**
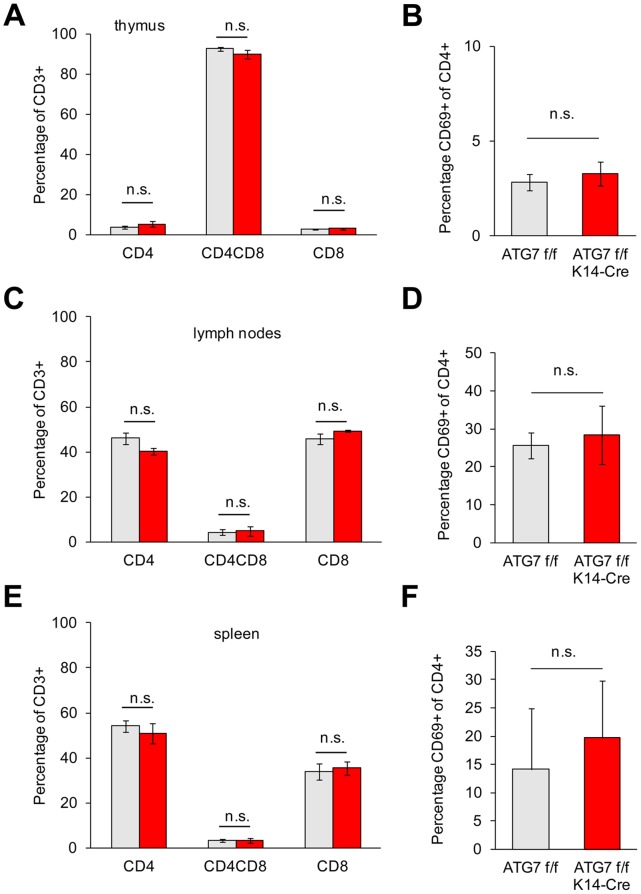
T cell differentiation into CD4+ and CD8+ cells and activation of CD4+ cells are not compromised in ATG7 f/f K14-Cre mice. Lymphocytes were prepared from the thymus (**A**, **B**), lymph nodes (**C**, **D**) and spleen (**E**, **F**) and subjected to FACS with antibodies against CD3, CD4, and CD8 (**A**, **C**, **E**) as well as with antibodies against CD4 and CD69 (**B**, **D**, **F**). Statistical analysis using the t-test showed that there were only non-significant (n.s.) differences between the genotypes. The results of one of two experiments with similar results are shown.

Next we investigated the relative abundance of lymphocyte populations in immune organs of the mice. Lymphocytes were prepared from the thymus, the lymph nodes and the spleen and subjected to FACS analysis using antibodies against CD45, CD3, CD4, CD8, and CD69. No significant differences in the relative abundance of T cells and B cells (not shown), CD4 and CD8 T cells, and activated CD4-positive cells in ATG7 f/f and ATG7 f/f K14-Cre were detected (Fig. 4).

### ATG7 f/f K14-Cre Mice do not Show a Higher Incidence of Tissue Inflammation than Normal Mice

The state of health of our colony of ATG7 f/f and ATG7 f/f K14-Cre mice was monitored over a period of more than 3 years. The mice did not show apparent signs of diseases. In contrast to mice receiving autophagy-deficient thymic transplants under the kidney capsule [12], female ATG7 f/f K14-Cre mice had fat pads of normal size (Fig. S3) and the diameter of the colon appeared normal in all mice investigated. A detailed study of the epidermis, in which the K14 promoter drives the deletion of ATG7 in keratinocytes, will be reported in a separate publication (Rossiter et al., manuscript in preparation).

**Figure 5:**
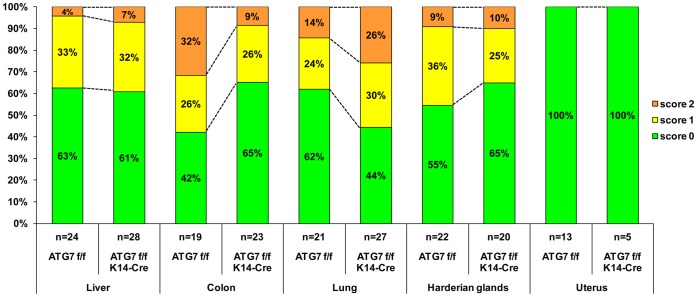
Tissue inflammation is not increased by suppression of autophagy in the thymic epithelium. The severity of inflammation in organs of mice expressing ATG7 (ATG7 f/f) or lacking ATG7 in the thymic epithelium was scored using H&E-stained sections. Scores 0 (green portion of the bars), 1 (yellow) and 2 (orange) mean no inflammation, mild and moderate inflammation (respectively). The bars show the percentage of mice with the respective score. The number of mice of each group is shown below the bars.

To evaluate tissue inflammation, potentially induced by autoreactive T cells [12], we performed H&E stainings of thin-sections of several organs. Only mild signs of inflammation were occasionally observed in young ATG7 f/f K14-Cre and control mice as well as in the corresponding mice of the GFP-LC3 transgenic mouse line (not shown). As we reasoned that potential effects of autoimmunity may become more pronounced with increasing age, we investigated large numbers of mice older than 6 months (Fig. 5). Organs in which the K14 promoter is active and thus drives the deletion of ATG7 f/f, contained either no inflammatory infiltrates (skin, uterus) or showed inflammation in approximately the same percentage of mice of both genotypes (Harderian glands) (Fig. 5). Likewise, organs in which the K14 promoter is inactive such as the colon, the lung and the liver contained inflammatory infiltrating cells in a similar fraction of ATG7 f/f and of ATG7 f/f K14-Cre mice (Fig. 5; Fig. S4). In all mice, the most severe cases of inflammation were much milder than those reported for mice receiving thymus transplants from autophagy-deficient embryos [12].

## Discussion

In this study we have tested the hypothesis that the molecular machinery of macroautophagy in the thymic epithelium is required to achieve negative selection of T cells. This hypothesis was put forward by Nedjic et al. [12] and was discussed in several recent review articles [6], [14], [15], [18], [19]. While Nedjic et al. had employed a technically sophisticated but artificial method of suppressing autophagy, our approach relied purely on genetic recombination. By transplanting the embryonic thymus under the kidney capsule of an adult mouse, Nedjic et al. generated a mouse completely devoid of ATG5-dependent autophagy in the entire thymus, whereas we suppressed ATG7-dependent autophagy specifically in the epithelium of the thymus. Specifically, our model depended on Cre-mediated recombination that is driven by the K14 promoter. Within the differentiated thymic epithelium of the adult mouse, K14 is expressed in the medulla but not in the cortex [20]. However, the K14 promoter is also active in the progenitor cells of the entire thymic epithelium [16] so that the Cre-mediated suppression of ATG7-dependent autophagy occurs in epithelial cells of both the thymic medulla and cortex. In line with this concept, the constitutive presence of GFP-LC3 labeled autophagosomes was abrogated in the epithelial cells of the cortex of ATG7 f/f K14-Cre mice. As the epithelium of the medulla of ATG7 f/f mice contained only very few green fluorescence puncta, which cannot be faithfully assigned to GFP-LC3-labeled autophagosomes in this part of the thymus [8], we obtained additional evidence for the blockade of autophagy in the medullary epithelium. Indeed, the epithelium of both the thymic cortex and the medulla of ATG7 f/f K14-Cre mice showed a diffuse cytoplasmic accumulation of GFP-LC3, suggesting that an otherwise constitutively ongoing autophagic degradation of GFP-LC3 was suppressed by K14-Cre driven deletion of ATG7 in both compartments of the thymus. Notably, the accumulation of GFP-LC3 was observed in virtually all TECs, indicating that the suppression of autophagy by the Cre-lox system was highly efficient. Moreover, p62 which is a widely used marker for autophagic flux [5], also accumulated strongly in the thymic epithelium of ATG7 f/f K14-Cre mice, confirming the efficient suppression of autophagy. Western blot analyses demonstrated not only the strong increase in the amounts of GFP-LC3 and p62 but also the prevention of LC3-II formation and the concomitant increase in the amount of endogenous LC3-I in the thymus of ATG7 f/f K14-Cre mice.

The main result of our study is that the deletion of ATG7-dependent autophagy in the thymic epithelium does not result in autoimmunity according to the criteria defined by Nedjic et al. [12]. Neither activation of CD4 T cells nor increased tissue inflammation nor clinically apparent disease manifestations leading to weight loss were observed in ATG7 f/f K14-Cre mice. These results argue against the hypothesis that autophagy in the thymic epithelium is essential for the establishment of self-tolerance as proposed by Nedjic et al. [12]. Several differences in the experimental design between our study and that of Nedjic et al. [12] may account for the apparent differences in the conclusions. Most obviously, the genetic targeting of the endogenous thymic epithelium as opposed to the transplantation of autophagy-deficient embryonic thymi is an important difference. In a control experiment of Nedjic et al. [12], treatment of embryonic thymus lobes with deoxyguanosine did not prevent autoimmunity in the recipient mice, which led the authors to conclude that carryover of ATG5-negative hematopoietic cells was not critical for disease development [12]. In the light of the results of our study, additional control experiments may be necessary to exclude effects of non-epithelial cells of the transplanted thymus. Other experimental differences include the genetic backgrounds of the mouse models, which were similar (both mainly C57BL/6) but not identical in our study and in that of Nedjic et al. [12], and the choice of the targeted gene. Since both ATG5 and ATG7 have essential roles in the same pathway of autophagy, the genetic approach of blocking autophagy *per se* is unlikely to explain the differences in the impact on the development of self-tolerance. However, an as yet unknown role of ATG5 outside of autophagy might cause gene-specific effects in the thymic epithelium.

What might be the function of constitutive autophagy in the thymic epithelium [8], [12] if it is not required for the suppression of autoimmunity? Apart from the potential role of autophagy in the positive selection of distinct CD4-positive T cell specificities [12] that was not addressed here, autophagy may contribute to cellular processes during thymic involution or during stress responses of the thymus. However, the lack of differences in the weight of the thymus of aged ATG7 f/f and ATG7 f/f K14-Cre mice argues against a critical role of ATG7-dependent autophagy in involution that was proposed by Uddin et al. [21]. Remarkably, high levels of autophagy appear to be a common feature of several epithelial cell types including those of the lens and the Harderian glands [8]. A common role of autophagy in all these epithelia is conceivable, yet elusive. Future studies in the ATG7 f/f K14-Cre mouse model may uncover the roles of autophagy in the epithelia of the thymus and of other organs.

In summary, this study establishes a model for the investigation of autophagy-dependent processes in thymus of fully immune-competent animals. As our screening for signs of autoimmunity was negative in this model, the hypothesis that autophagy within the thymic epithelium is required for self-tolerance could not be supported here.

## Materials and Methods

### Ethics Statement

The animal studies were approved by the Ethics Review Committee for Animal Experimentation of the Medical University of Vienna, Austria (approval number BMWF-66.009/0124-II/10b/2010).

### Mice

ATG7-floxed mice and GFP-LC3 transgenic mice have been described previously [10], [8]. K14-Cre mice (strain Tg(KRT14-cre)1Amc/J) were obtained from the Jackson Laboratory, Bar Harbor, MN. K14-Cre mice were crossed with ATG7-floxed mice to yield ATG7 f/f and ATG7 f/f K14-Cre mice which were housed under specific pathogen-free conditions. Crossing the GFP-LC3 transgene into these mice yielded GFP-LC3 ATG7 f/f and GFP-LC3 ATG7 f/f K14-Cre mice that were housed in a conventional animal facility. All mice were maintained in a regular 12 hours light-dark cycle on a normal diet with free access to drinking water (Rossiter et al., manuscript in preparation).

### Weight of Body, Thymus and Spleen

Immediately after the mice were sacrificed, the body weight was measured. Thymus and spleen were prepared and weighed. The weights and the ratio of thymus weight to body weight as well as the ratio of spleen weight to body weight were compared between groups of ATG7 f/f and ATG7 f/f K14-Cre mice of the same sex and age. The values were analyzed using the t-test, and p-values smaller than 0.05 were considered significant.

### Fluorescence and Immunofluorescence Analysis

For the analysis of the fluorescence of GFP-LC3 *in situ*, mice were anaesthetized and perfused through the left ventricle with 4% paraformaldehyde in phosphate-buffered saline (PBS). The thymus of each mouse was collected and further fixed with the same fixative for 4 h, followed by treatment with 15% sucrose in PBS for 4 h at room temperature and then with 30% sucrose solution overnight at 4°C. Tissue samples were embedded in optimal cutting temperature (OCT) medium and stored at −80°C. For the investigation of other non-GFP-LC3 samples, mice were sacrificed and thymus tissues were fixed in phosphate-buffered 7.5% formaldehyde for 24 h and embedded in paraffin. The samples were sectioned at 5 µm thickness. Sections of paraffin-embedded samples were pretreated by microwave heating in Target Retrieval Solution (DakoCytomation, Glostrup, Denmark) according to a protocol published previously [22]. Rat anti-keratin 8 (K8) diluted 1∶800 (TROMA-1, Developmental Studies Hybridoma Bank, Iowa City, IA), polyclonal rabbit anti-keratin 5 (K5) (Covance) diluted 1∶1000, and rabbit polyclonal anti-p62 (Enzo Lifescience, Vienna, Austria) antibodies diluted 1∶2000 in PBS, pH 7.2, 2% bovine serum albumin (BSA) were applied overnight at 4°C. The samples were then incubated with 10% goat serum (DakoCytomation) to block non-specific binding of second step antibodies, and finally with goat anti-rat or goat anti-rabbit antisera conjugated with Alexa Fluor 546 or 488 (Molecular Probes, Leiden, The Netherlands) for 30 minutes. Hoechst 33258 (Molecular Probes) was used to label the nuclei. The presence of GFP-LC3 puncta was investigated using an LSM700 confocal laser microscope (Zeiss), other studies were done with a conventional fluorescence microscope.

### Fluorescence-activated Cell Sorting (FACS) Analysis of Lymphocytes

Mouse thymus, lymph nodes (1 axillary and 1 inguinal lymph node of each mouse were combined) and spleen were dissected and passed through a 40 µm cell strainer (BD Biosciences). The passed cells were washed with PBS and remaining erythrocytes were lysed with a commercially available hemolysis buffer (Morphisto, Frankfurt am Main, Germany). Cells were washed and analyzed using the following fluorescence-labeled monoclonal antibodies: fluorescein isothiocyanate (FITC)-anti-CD45, peridinin chlorophyll protein (PerCP)-anti-CD3, FITC-anti-CD4, phycoerythrin (PE)-anti-CD8, PE-anti-CD19, PerCP-anti-CD69 and PE-anti-CD62L. All antibodies were obtained from Biozyme (Oldendorf, Germany). Appropriate isotype controls were included and gates were set according to isotype-matched controls. Analysis of cells was performed on a FACSCalibur flow cytometer (BD Biosciences), and data were evaluated using the FlowJo software (Tree Star, Ashland, OR).

### Western Blot

Thymi were placed in lysis buffer containing 50 mM Tris (pH 7.4), 2% SDS and complete protease inhibitor cocktail (Roche, Mannheim, Germany) and the tissue was dissociated by sonication. The insoluble debris was removed by centrifugation and the protein concentration of the supernatant was measured by the BCA (bicinchoninic acid) method (Pierce, Rockford, IL). Western blot analysis was performed as described previously [23]. The following first step antibodies were used for the detection of specific antigens: rabbit polyclonal anti-p62 (Enzo Lifescience, 1∶2000), rabbit polyclonal anti-LC3 (1∶2000, GeneTex, Irvine, CA) and rabbit polyclonal anti-GFP (Abcam, Cambridge, UK, 1∶6000).

### Histopathology

Immediately after mice were sacrificed, the organs were collected and fixed in phosphate-buffered 7.5% formaldehyde. Tissue sample were embedded in paraffin, thin-sectioned at a thicknes of 5 µm, stained with H&E, and screened for signs of inflammation [24]. The scoring of tissue inflammation was done by an experienced pathologist in a blinded manner with regard to the genotype of the mice. Blinded scoring by a second investigator yielded essentially the same results. Scores 0, 1, and 2 indicated the absence of inflammatory infiltrates, the presence of small inflammatory infiltrates, and the presence of large inflammatory infiltrates, respectively. Higher scores for “manifest signs of tissue destruction” [12] were considered, however, samples with such a severe phenotype were not observed.

## Supporting Information

Figure S1 **The weights of the body, thymus and spleen of female ATG7 f/f K14-Cre mice are normal.** The weights of the total body (**A**), the thymus (**B**) and the spleen (**C**) were determined for female mice in the age range from 5 to 12 months. The weight data of mice expressing ATG7 (ATG7 f/f) (n = 5) or lacking ATG7 (ATG7 f/f K14-Cre) (n = 6) in the thymic epithelium were compared in absolute numbers or relative to the body weight (**D**, **E**). Statistical analysis using the t-test showed that there were only non-significant (n.s.) differences between the genotypes.
(PDF)

Figure S2 **The thymus of ATG7 f/f K14-Cre mice is morphologically normal.** Thin-sections of the thymus of mice aged 2 months (**A**) and 20 months (**B**) were stained with H&E. (**C**) Double-immunolabeling of the thymus (age 5 months) with antibodies against K5 (green) and K8 (red) showed the same pattern in ATG7 f/f and ATG7 f/f K14-Cre mice. Nuclei are labeled with Hoechst 33258 (blue) in the lower panels. C, cortex; M, medulla. The complete field of view under 200-fold magnification is shown in all panels.
(PDF)

Figure S3 **The amount of fat tissue is normal in ATG7 f/f K14-Cre mice.** Arrows point to the fat of female mice.
(PDF)

Figure S4 **Tissue inflammation is not significantly increased in ATG7 f/f K14-Cre** **mice.** Thin-sections of sections of ATG7 f/f and ATG7 f/f K14-Cre mice were stained with H&E. Exemplary results are shown. Note that there was significant variation in each groups (ATG7 f/f and ATG7 f/f K14-Cre), as summarized in Figure 5. In particular, the areas of the cross-sections of the uterus (lowermost panels) varied but there were no consistent differences between ATG7 f/f and ATG7 f/f K14-Cre mice. The photos show the complete field of view under 40-fold (colon, uterus) or 100-fold (liver, lung) magnification. Tissue areas containing inflammatory infiltrates are marked with arrows.
(PDF)
