# Supplementary figures and images for: Autophagy in the Thymic Epithelium Is Dispensable for the Development of Self-Tolerance in a Novel Mouse Model
Source: PLoS One. 2012 Jun 18;7(6):e38933. doi: 10.1371/journal.pone.0038933 (PMC3377705; doi:10.1371/journal.pone.0038933)

**A**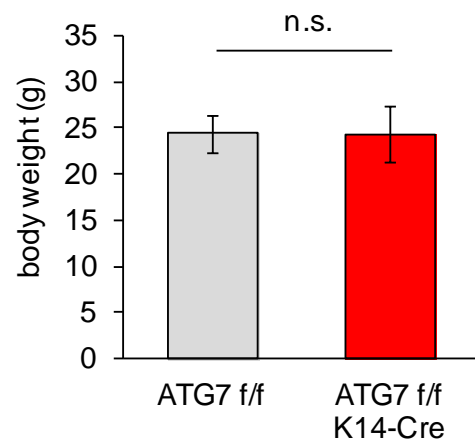**B**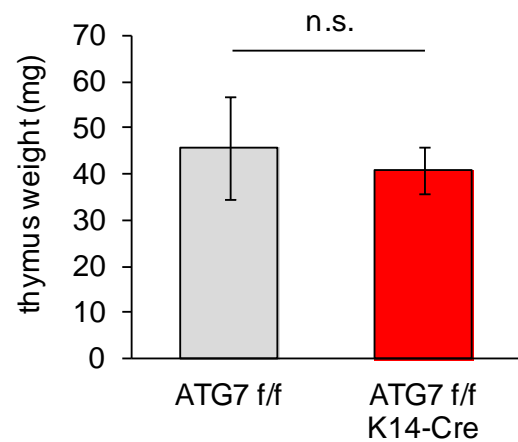**C**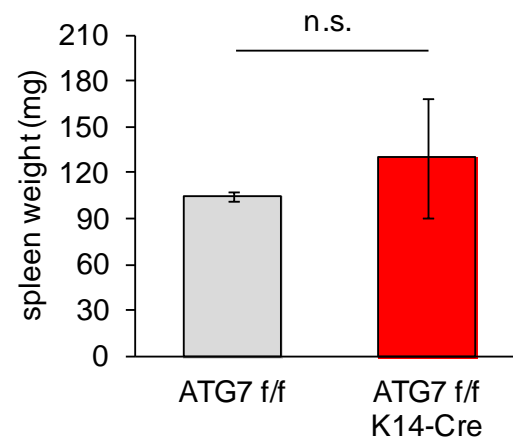**D**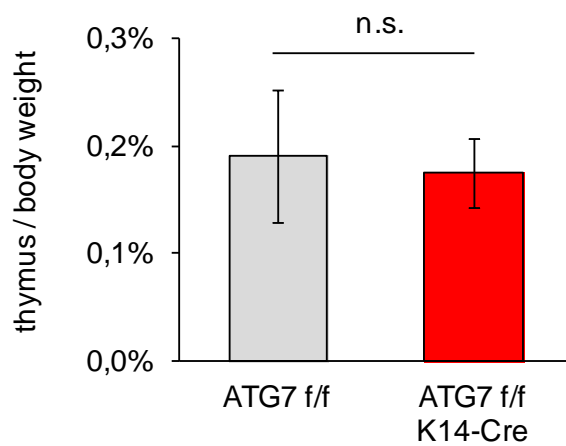**E**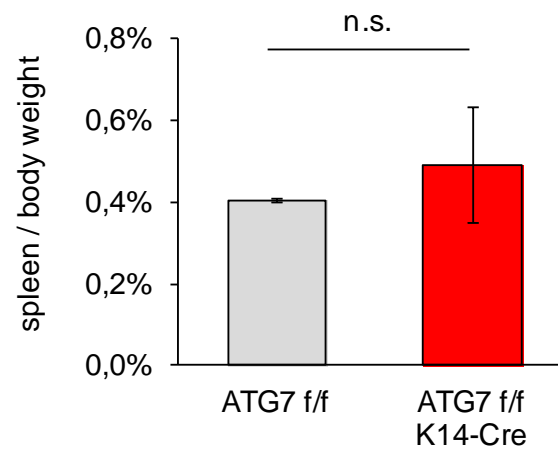

Supplement: Figure S1 — The weights of the body, thymus and spleen of female ATG7 f/f K14-Cre mice are normal. The weights of the total body (A), the thymus (B) and the spleen (C) were determined for female mice in the age range from 5 to 12 months. The weight data of mice expressing ATG7 (ATG7 f/f) (n = 5) or lacking ATG7 (ATG7 f/f K14-Cre) (n = 6) in the thymic epithelium were compared in absolute numbers or relative to the body weight (D, E). Statistical analysis using the t-test showed that there were only non-significant (n.s.) differences between the genotypes. (PDF) [file pone.0038933.s001.pdf]

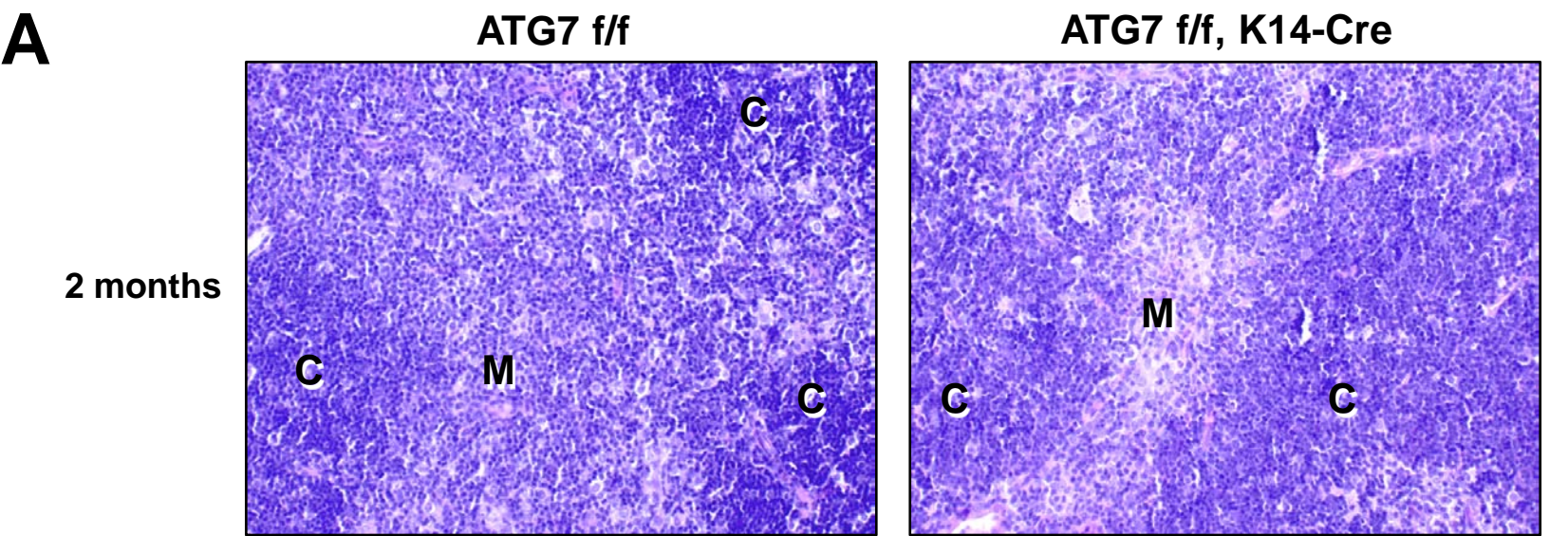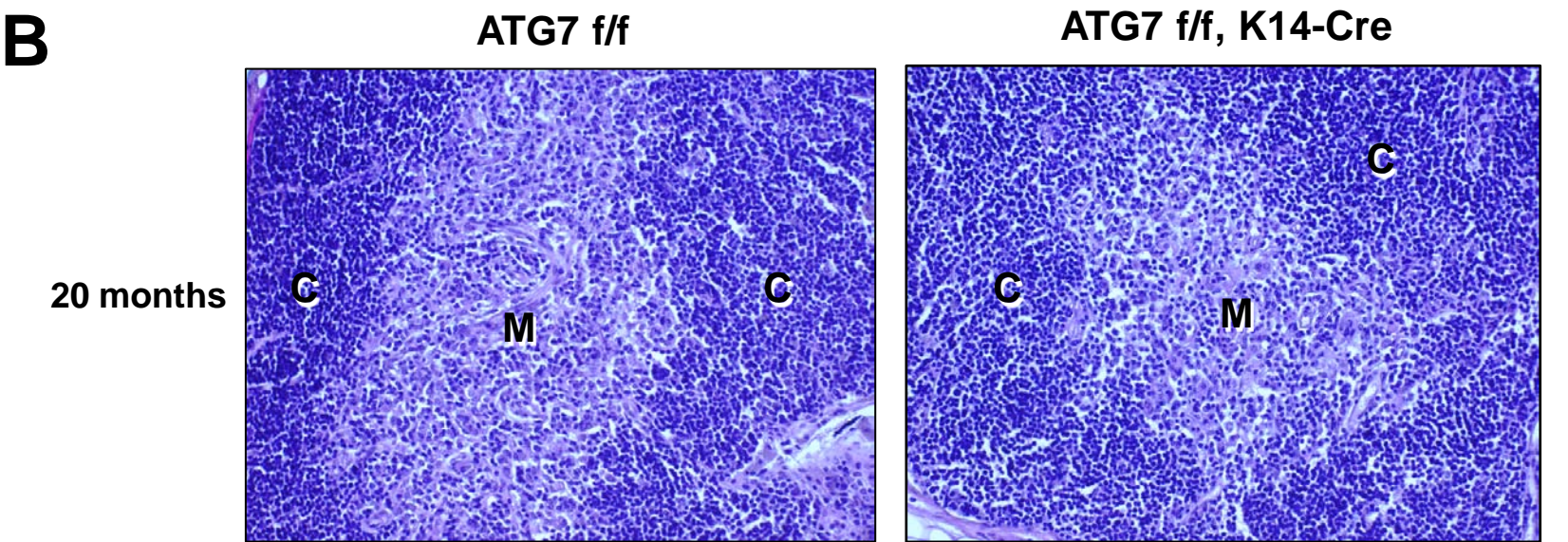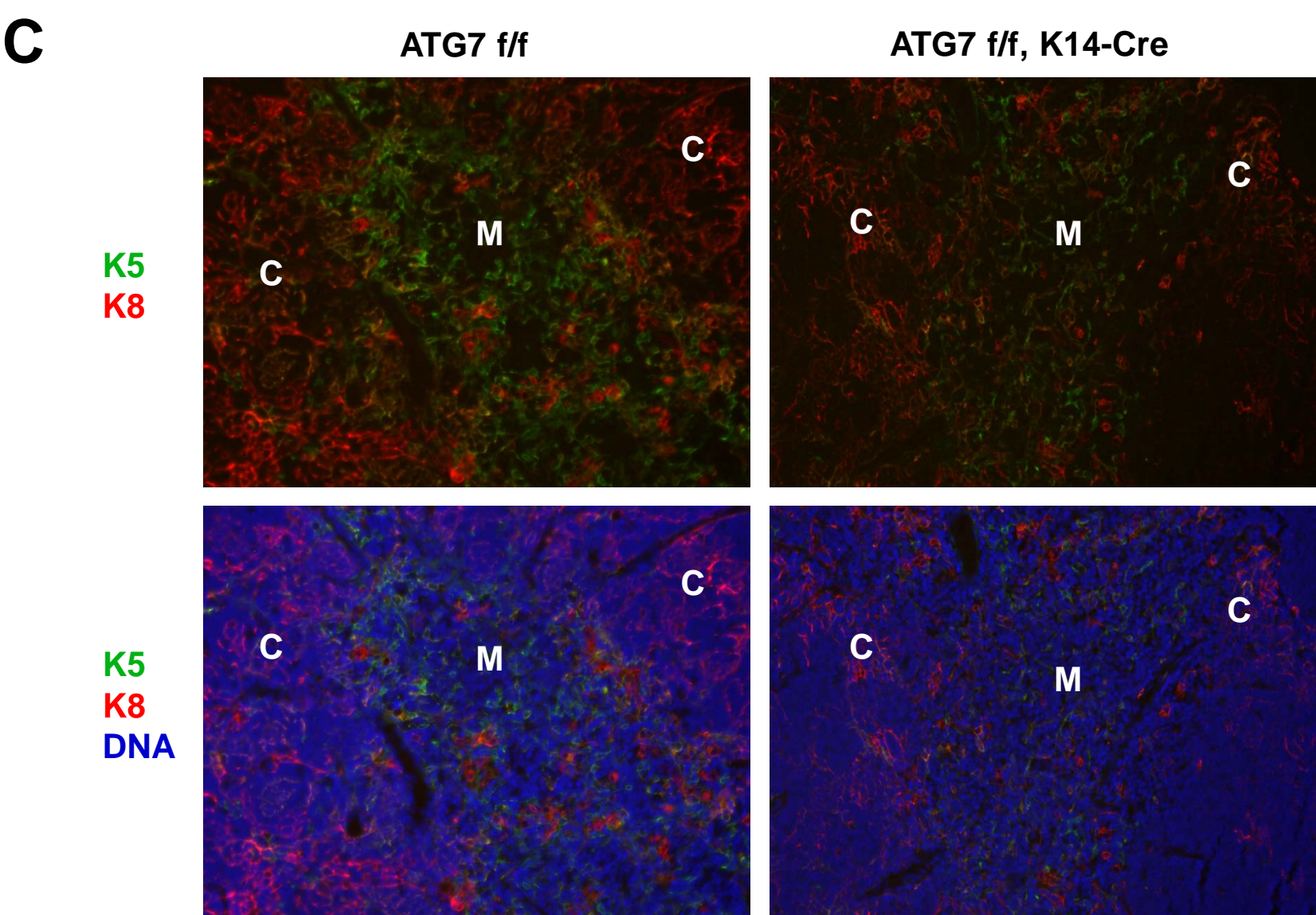

Supplement: Figure S2 — The thymus of ATG7 f/f K14-Cre mice is morphologically normal. Thin-sections of the thymus of mice aged 2 months (A) and 20 months (B) were stained with H&E. (C) Double-immunolabeling of the thymus (age 5 months) with antibodies against K5 (green) and K8 (red) showed the same pattern in ATG7 f/f and ATG7 f/f K14-Cre mice. Nuclei are labeled with Hoechst 33258 (blue) in the lower panels. C, cortex; M, medulla. The complete field of view under 200-fold magnification is shown in all panels. (PDF) [file pone.0038933.s002.pdf]

**ATG7 f/f**

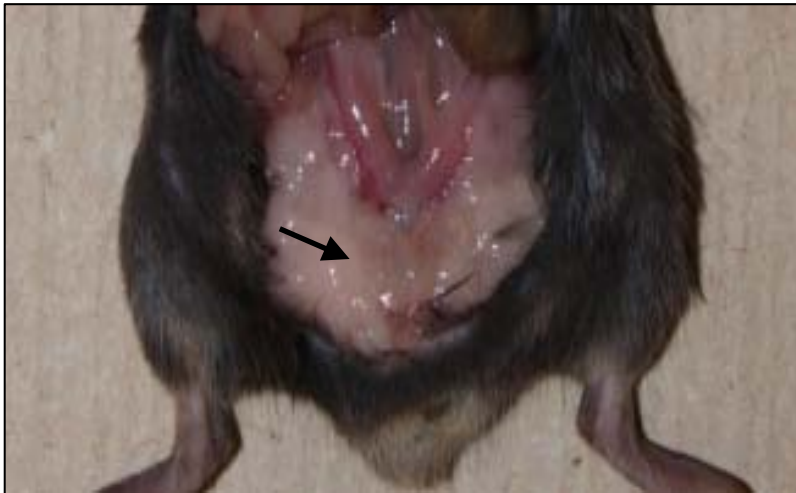

**ATG7 f/f, K14-Cre**

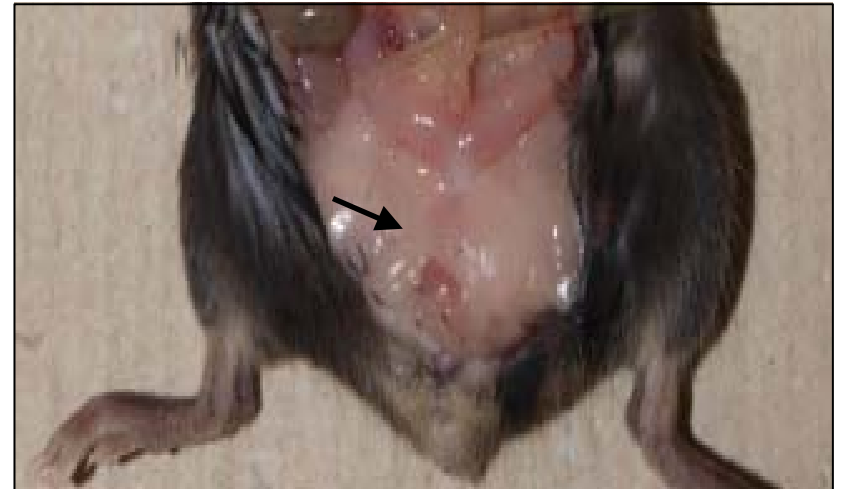

Supplement: Figure S3 — The amount of fat tissue is normal in ATG7 f/f K14-Cre mice. Arrows point to the fat of female mice. (PDF) [file pone.0038933.s003.pdf]

**ATG7 f/f**

**ATG7 f/f, K14-Cre**

**Colon**

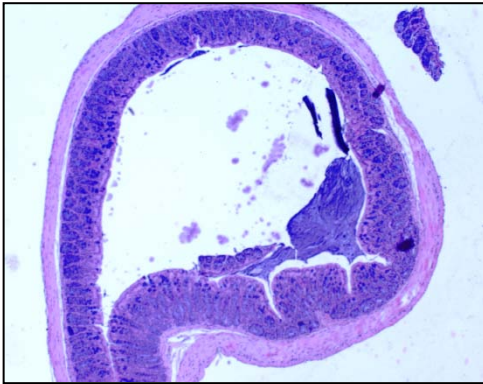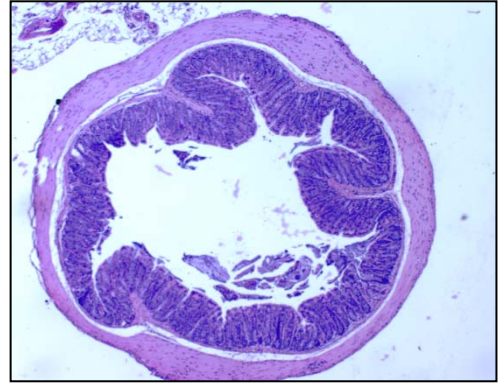

**Liver**

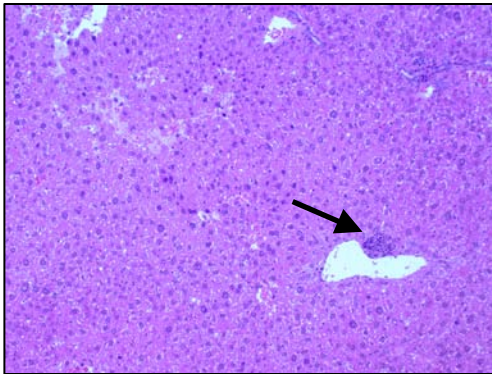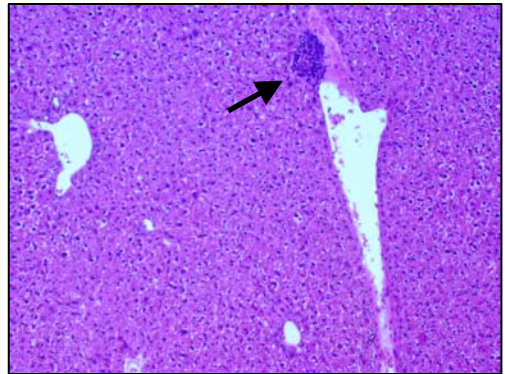

**Lung**

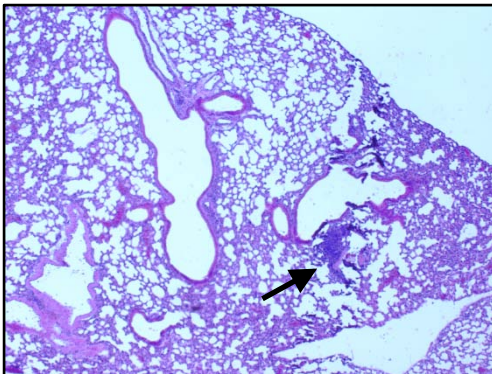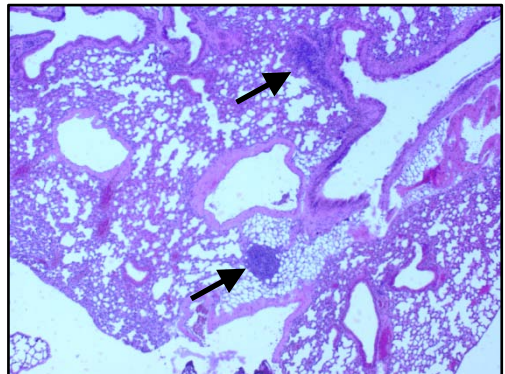

**Uterus**

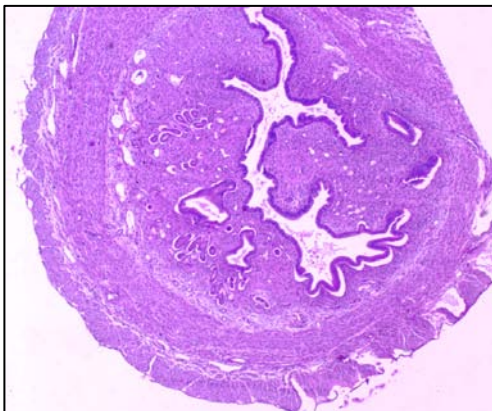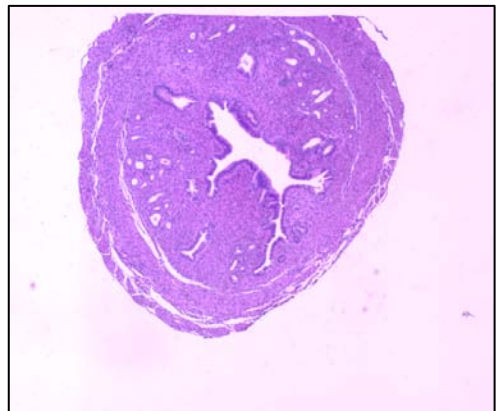

Supplement: Figure S4 — Tissue inflammation is not significantly increased in ATG7 f/f K14-Cre mice. Thin-sections of sections of ATG7 f/f and ATG7 f/f K14-Cre mice were stained with H&E. Exemplary results are shown. Note that there was significant variation in each groups (ATG7 f/f and ATG7 f/f K14-Cre), as summarized in Figure 5. In particular, the areas of the cross-sections of the uterus (lowermost panels) varied but there were no consistent differences between ATG7 f/f and ATG7 f/f K14-Cre mice. The photos show the complete field of view under 40-fold (colon, uterus) or 100-fold (liver, lung) magnification. Tissue areas containing inflammatory infiltrates are marked with arrows. (PDF) [file pone.0038933.s004.pdf]
